# Research on Plant Genomics and Breeding: 2023

**DOI:** 10.3390/plants13212998

**Published:** 2024-10-26

**Authors:** Zhihui Chen, Xiaohong Tong, Jian Zhang, Jie Huang, Zhiyong Li

**Affiliations:** 1 Tea Research Institute of Fujian Academy of Agricultural Sciences, Fuzhou 350013, China; chenzhihui75@sina.com; 2 State Key Laboratory of Rice Biology and Breeding, China National Rice Research Institute, Hangzhou 311400, China; tongxiaohong@caas.cn (X.T.); zhangjian@caas.cn (J.Z.)

Over the past two decades, the rapid progress made in plant breeding has been significantly driven by the integration of knowledge in the fields of plant genomics and genetics, and by the application of state-of-the-art biotechnologies. Furthermore, collaborative initiatives within community-based projects have yielded substantial contributions to these advancements. This article aims to furnish a comprehensive overview of recent publications in *Plants*, with a specific emphasis on a diverse array of molecular biology research methodologies associated with plant genomics and breeding. 

## 1. Genetic Basis Underpinning Plant Trait

Amalova and colleagues from the Institute of Plant Biology and Biotechnology, Kazakhstan, attempted to identify Quantitative Trait Loci (QTLs) associated with wheat adaptation traits using a Nested Association Mapping (NAM) population [1]. The authors evaluated plant adaptation-related traits in two contrasting regions, including the heading date, the seed maturation date, the plant height, the peduncle length, and the yield of 290 recombinant inbred lines (RILs) of the NAM population. The integration of findings from 10,448 polymorphic SNP (single-nucleotide polymorphism) markers in the genome-wide association study (GWAS) led to the identification of 74 QTLs associated with four agronomic traits under study. Notably, 16 of these QTLs had been consistently identified in previous studies. To date, this study represents the inaugural analogous investigation in terms of applying NAM to cereals in Kazakhstan. It yielded a valuable data source for the identification of novel genes associated with the adaptation of wheat plants.

Anther morphology, particularly anther length, is genetically controlled by QTLs. Researchers have detected QTLs for anther length in different populations, but no candidate genes have been identified despite the critical role of this parameter in determining hybrid rice seed production. A research team from the China National Rice Research Institute reported the fine mapping of *qAL5.2* for anther size using backcross inbred lines (BILs) [2]. Using gene chip technology, the authors identified six *qALs*, including *qAL5.1* and *qAL5.2*, which explained 35.73% and 25.1% of the phenotypic variance in the population, respectively. *ORF8* encoded a lipase belonging to the alpha/beta-hydrolase (ABH) family. It is a likely candidate for *qAL5.2* and is possibly involved in regulating alpha-linolenic acid metabolism, which controls anther length. 

Similarly, Yin et al. (2024) analyzed the genetic basis of yield traits in rice and validated a novel QTL for grain width and weight. Nine yield-related traits were investigated using an F2 population containing 190 plants [3]. The authors discovered that grain yield per plant was positively correlated with six yield traits, except for grain length and width. The highest correlation coefficient—0.98—was observed with regard to the number of filled grains per plant. Simultaneously, a linkage map covering the whole genome was constructed by genotyping each plant with the selected polymorphic markers using the F_2_ population. All told, 36 QTLs for the yield traits were detected on nine chromosomes, and the phenotypic variation was explained by a single QTL that ranged from 6.19% to 36.01%. The identification of a major QTL for grain width and weight, denoted as *qGW2-1*, was refined to an interval of approximately 2.94 megabases. Upon comparative analysis of QTL locations, it was observed that no previously reported QTLs for grain width (GW) and thousand grain weight (TGW) shared an overlapping interval with *qGW2-1*. This suggests that *qGW2-1* represents a novel QTL, with substantial potential for application in molecular design breeding strategies for rice.

Kenaf (*Hibiscus cannabinus* L.), originating from Africa, is an essential crop for fiber and other industrial materials, but its genetics have yet to be explored. A recent publication in *Plants* reported the GWAS analysis of seven agronomic traits in Kenaf: days to flowering, plant height, fresh weight, dry weight, flower color, stem color, and leaf shape [4]. In a cohort of 96 germplasm accessions, encompassing 55 gamma irradiation-derived mutant lines, an analysis using genotyping by sequencing (GBS) revealed the presence of 49,241 single-nucleotide polymorphisms (SNPs). Of these, 49 SNPs are associated with important agronomic traits, such as days to flowering, plant height, fresh weight, dry weight, flower color, stem color, and leaf shape. Except for fresh and dry weights, no single-nucleotide polymorphisms (SNPs) were detected in the genic region. Most SNPs were identified within the exon of the respective genic regions. The identified genes exhibit a homology of 45% to 96% with plants of the Malvaceae and Betulaceae families and are known to play a role in plant growth and development through various pathways. These findings are anticipated to enhance selective breeding programs for Kenaf, thereby improving overall plant quality.

As integral constituents of the SCF complexes, F-box proteins play pivotal roles in responding to both abiotic and biotic stresses in plants. The wheat F-box protein, TaFBA1, has been recognized for its reactivity to abiotic stress in tobacco. Nonetheless, its precise biological functions within wheat itself are yet to be fully elucidated. Using transgenic wheat with enhanced and suppressed expression of *TaFBA1*, Li et al. (2024) found that *TaFBA1* is a positive regulator of drought tolerance [5]. Compared to WT and RNAi lines, OE plants showed a higher survival rate and fresh weight and a more robust antioxidant capacity, as well as the maintenance of stomatal openings. *TaFBA1* is implicated in the regulation of genes associated with antioxidants, fatty acid, and lipid metabolism, as well as with cellulose synthesis. Consequently, this study yields a valuable gene reservoir for use enhancing abiotic stress tolerance in crops through genetic interventions.

The proteins responsible for reading DNA methylation can specifically adhere to methylated CpG dinucleotides via methyl-CpG-binding domains (MBDs) or SET- and ring-finger-associated domains. Presently, the *Arabidopsis thaliana* genome has revealed the existence of at least thirteen AtMBD proteins, although the functions of certain members remain ambiguous. A recent publication in *Plants* presented the functional characterization of *AtMBD3* [6]. The *AtMBD3* gene exhibits high expression levels in pollen and seeds, displaying a preference for binding to methylated CG and CHG and to unmethylated DNA sequences. Consistent with its murine ortholog, *mbd3* mutants demonstrated reduced mature pollen production and experienced early- or late-stage embryo abortion, likely stemming from asymmetric divisions. Transcriptome analysis of the seeds revealed the differential expression of numerous key transcription factors involved in embryo development. Moreover, in yeast and in vivo, a pollen-specific protein, PBL6, was discovered to interact with AtMBD3. Hence, this study offers significant functional insights into AtMBD3, contributing to a more comprehensive understanding of the AtMBD family.

## 2. Gene Function Studies Using RNA Molecules

RNA interference (RNAi) has been found to trigger genemic epigenetic modifications, effectively regulating gene expression in plants. RNAi constitutes a regulatory and defensive mechanism, pivotal in the growth, development, and management of plant responses to pathogens and abiotic stresses. In the context of spray-induced gene silencing (SIGS), the force known as exogenous RNA interference, complementary to target plant genes, reduces the expression of said target genes. In their study, Suprun et al. (2024) examined the influence of specific exogenous dsRNAs on the promoter, coding sequence (CDS), and intron regions of the *SlTRY* tomato gene, which encodes an R3-type MYB repressor of anthocyanin biosynthesis [7]. Their findings indicate that achieving efficient gene silencing in plants necessitates the targeting of a protein-coding region with dsRNA of at least 50 base pairs in length, exemplified by dsRNA-Prom2. Interestingly, dsRNA-Intron treatment increased the expression of the *SlTRY* gene compared to the control under normal growth conditions. The suppression of the *SlTRY* gene increased the concentration of anthocyanins and led to the simultaneous elevation of other compounds within the phenylpropanoid pathway. This research underscores the efficacy of dsRNA as a potent instrument for expeditiously probing gene functionality in plant science.

As mentioned above, the application of double-stranded RNAs (dsRNAs) to plant surfaces has been greatly beneficial in plant biotechnology and gene function studies. However, the comprehensive impact of dsRNA, particularly on the intricate network of microRNAs (miRNAs), remains unclear. Nityagovsky and colleagues tested the effects of the exogenous application of chalcone synthase (*CHS*)-encoding dsRNA in terms of altering the miRNA transcriptome in the rosette leaves of Arabidopsis thaliana [8]. Compared to the non-specific dsRNA control, a two-day treatment of *AtCHS*-dsRNA induced alterations in the expression pattern of 59 miRNAs. These miRNAs can be categorized into 17 major gene ontology (GO) groups, notably including the aromatic compound biosynthesis pathway associated with *CHS* activity. qRT-PCR results verified that the transcription of the predicted targets of DEG miRNAs changed accordingly, implying a negative correlation between the expression of miRNAs and their predicted targets. Thus, exogenous plant gene-specific dsRNAs induce substantial changes in the plant miRNA composition, ultimately affecting the expression of a wide range of genes. These findings have profound implications for our understanding of the effects of exogenously induced RNA interference, which can have broader effects beyond targeted mRNA degradation. Through the lens of miRNA profile, this work revealed that gene-specific dsRNAs induce specific and massive cellular responses at the epigenetic level after exogenous application.

Short interfering RNAs (siRNAs) are converted by dsRNAs with specialized enzymes, known as dicer-like enzymes, and are integrated into RNA-induced silencing complexes to effectively inhibit gene expression in plants. The work of Kiselev et al. (2024) found that simultaneous foliar application of gene-specific dsRNAs can decrease gene expression in response to activating anthocyanin accumulation in *Arabidopsis thaliana* [9]. They discovered that a combination of external dsRNAs or siRNAs, administered at two different doses, could silence the expression of the target gene, leading to a more effective increase in anthocyanin content than using a single dsRNA treatment. 

## 3. Using OMICs to Understand Key Biological Process in Plants

The presence of a “beany flavor” in soybeans represents an unfavorable characteristic that diminishes their market value. Dongfudou 3 is a highly coveted soybean variety because it lacks this undesirable trait. However, the absence of the genome sequence of Dongfudou 3 has hindered the investigation into the genes responsible for this beany flavor. Duan and colleagues, originating from China, conducted a genomic survey of a distinct soybean variety utilizing next-generation sequencing technology [10]. The survey revealed an estimated genome size of approximately 1.07 Gb, with 72.50% of this comprising repetitive sequences. The 916.00 Mb draft genome sequence was successfully anchored onto 20 chromosomes, housing 46,446 genes and 77,391 transcripts. Notably, employing the principles of collinearity and sequence similarity, three genes, namely, *GmLox1*, *GmLox2*, and *GmLox3*, were identified and mapped within the Dongfudou 3 genome. Mutations in these genes significantly reduced transcript abundance and enzyme activity, potentially contributing to the absence of a beany flavor in Dongfudou 3. Utilizing third-generation sequencing, this study not only facilitates the formulation of advanced strategies for constructing a high-quality genome of Dongfudou 3, but also provides a preliminary comprehension of specific characteristics of Dongfudou 3 through the assembled draft genome. 

Plant-derived bioactive compounds, specifically glucosinolates (GSLs), play a crucial role in human health. A research team from Korea conducted a comprehensive analysis of 17 glucosinolate compounds in leaf samples obtained from 65 pak choy germplasm accessions using an acuity ultra-high-performance liquid chromatography (UHPLC) analytical system [11]. The variation in GSL compounds led to the categorization of the pak choy germplasm accessions into three distinct groups. This classification has promising prospects for use in future breeding endeavors seeking to enhance the glucosinolate content within the crop.

The biosynthesis of anthocyanin, an antioxidant with functional food properties for humans, is significantly influenced by light availability. Danpreedanan et al. (2023) conducted a study to examine the impact of shading on the yield and anthocyanin content of purple rice [12]. The findings revealed that increased shading resulted in higher anthocyanin content in the grain pericarp while concurrently reducing yield. The four tested rice varieties displayed varying responses to shading treatment in terms of grain yield, leaf chlorophyll content, and anthocyanin content. Phenotypical variation was observed to be independent of leaf colors. Although it has already been established that the *OsDFR* gene leads to a red grain color, the expression level of the *OsDFR* gene showed a weak correlation with anthocyanin content under shading stress, suggesting the involvement of other genes in anthocyanin biosynthesis when light intensity is low. This study indicates that certain varieties of purple rice can thrive and produce satisfactory yields under low-light conditions. These findings could potentially be valuable for the commercial cultivation of purple rice when employed in an optimal shading technique to enhance the antioxidant content of purple rice grains.

In addition to synthesizing metabolites, light intensity can significantly affect fruit development. A study by Gao et al. (2023) found that in watermelon, low-light stress led to a decrease in fruit size and the content of soluble sugar and amino acids compared to the control [13]. Fruits at 0–15 days after pollination were very sensitive to shading in terms of fruit expansion. The authors conducted transcriptome analysis at four stages and identified 8837 differentially expressed genes (DEGs), including 55 DEGs related to oxidation reduction, secondary metabolites, carbohydrate and amino acid metabolism, and transcriptional regulation. This study lays the groundwork for further research into the functions of low-light-stress-responsive genes and the molecular mechanisms underlying the effects of low-light stress on watermelon fruit expansion. 

## 4. Conclusions and Perspectives

Plant genomics and breeding play crucial roles in terms of addressing the global challenges of food security, climate change, and sustainable agriculture. By studying the genetic makeup of plants, scientists can identify genes responsible for desirable traits such as high yield, disease resistance, and tolerance to environmental stresses. In summary, this Editorial thoroughly reviewed the 13 research articles that were recently published in the Special Issue of *Plants* entitled “*Research on plant genomics and breeding 2023*”. These studies represent the latest progress in the genetic basis exploration of major agronomic traits using traditional genetic mapping or modern genotype-to-phenotype association technologies and cover various plant species like rice, wheat, Kenaf, and *Arabidopsis*. Employing genomics, transcriptomics, and metabolomics technologies, scientists gained deep insights into the biological puzzles of fruit development and beneficial metabolite biosynthesis. Additionally, researchers investigated the intricacy of SIGC in terms of target sites, dsRNA interaction, and effects on miRNA profiles. Overall, integrating plant genomics and breeding offers promising opportunities to enhance crop productivity, nutritional quality, and resilience, ultimately contributing to global food security and environmental sustainability. 

## Data Availability

No new data were created or analyzed in this study. Data sharing is not applicable to this article.
